# Advancements in Quasi-Solid-State Li Batteries: A Rigid Hybrid Electrolyte Using LATP Porous Ceramic Membrane and Infiltrated Ionic Liquid

**DOI:** 10.1021/acsaem.3c02828

**Published:** 2024-02-06

**Authors:** Deborath M. Reinoso, Carmen de la Torre-Gamarra, Antonio J. Fernández-Ropero, Belén Levenfeld, Alejandro Várez

**Affiliations:** † Departamento de Ciencia e Ingeniería de Materiales e Ingeniería Química, Universidad Carlos III de Madrid, Avda. Universidad 30, Leganés 28911, Spain; ‡ Instituto de Química del Sur (INQUISUR), CONICET, Departamento de Química, Universidad Nacional del Sur (UNS), Avda. Alem 1253, Bahía Blanca 8000, Argentina

**Keywords:** hybrid electrolytes, quasi-solid-state electrolyte, porous ceramic support, ionic liquid, Li battery

## Abstract

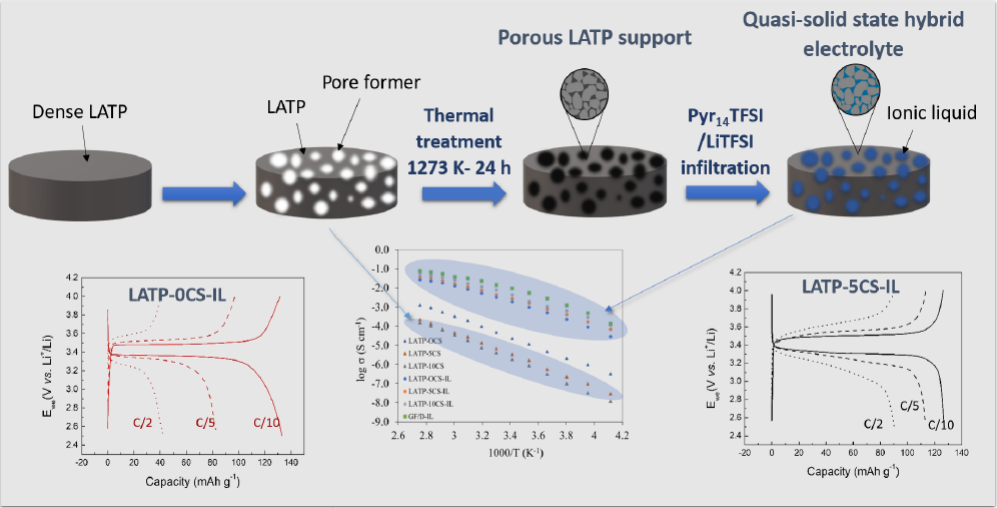

Despite the progress made in Li-ion battery components, technology still faces major challenges. Among them, the development of novel electrolytes with promising characteristics is required for next-generation energy storage devices. In this work, rigid hybrid electrolytes have been prepared by infiltration of an ionic liquid solution (Pyr_14_TFSI) with a lithium salt (LiTFSI) into a sintered LATP ion-conducting porous ceramic. The porous ceramic 3D network was obtained via solid-state sintering of LATP powders mixed with a small amount of corn starch as pore former. A synergetic effect between the ionic liquid and support was evidenced. The resultant quasi-solid-state hybrid electrolytes exhibit high ionic conductivity (∼10^–3^ S·cm^–1^ at 303 K), improved ion transfer number, *t*_*Li*_+, and a wide electrochemical window of 4.7–4.9 V vs Li^+^/Li. The LATP porosity plays a critical role in the free Li^+^ charge because it favors higher TFSI^–^ confinement in the ceramic interfaces, which consequently positively influences *t*_*Li*_+ and ionic conductivity. Electrochemical tests conducted at room temperature for Li/LiFePO_4_ cells using the hybrid electrolyte exhibited a high capacity of 150 mAh·g^–1^_LFP_ at C/30, and still retained 60 mAh·g^–1^_LFP_ at 1 C, while bare LATP does not perform well at low temperatures. These findings highlight this hybrid electrolyte as a superior alternative to the ceramic LATP electrolyte and a safer option compared with conventional organic electrolytes.

## 1 Introduction

Lithium-ion batteries (LB) present higher energy density, longer cycle life (larger number of charge/discharge cycles), lighter weight, and lower lost load (self-discharge) than other conventional energy storage systems. Nevertheless, the current electronic technologies and electric vehicle market expansion need the development of cost-effective, consumer-safe, and high-performance lithium batteries. Thus, the all-solid-state battery (ASSB) employing solid or quasi-solid electrolytes emerges as a promising alternative that allows overcoming safety concerns and offers higher energy densities. In recent years, great efforts to implement ASSB as a feasible energy storage system have been made.^1^ Accordingly, current research is focused, among other topics, on developing advanced electrolytes with high ionic conductivity, high electronic resistance, high cation-transference number, wide electrochemical stability window, outstanding thermal stability, and reduced interfacial resistance.^2^

Ionic liquids (ILs) are typically hydrophobic, nonflammable, and thermally stable. Moreover, ILs-based electrolytes containing Li^+^ salt provide high thermal stability, good ionic conductivity at room temperature, and a wide electrochemical window.^3,4^ Additionally, ILs offer high compatibility with lithium metal.^5,6^ In this regard, Chen et al.^7^ reported enhanced Li-battery cycling stability employing 0.2 LiTFSI/0.8 N-butyl-*N*-methylpyrrolidinium bis(fluoromethanesulfonyl) imide (Pyr_14_FSI), which reaches a capacity retention above 85% for 2000 cycles at 1 C. However, the ILs-based electrolytes cannot avoid electrolyte leakage and chiefly show a low lithium-ion transference number (*t*_*Li*_+< 0.1) indicating the low contribution of Li^+^ species to the overall charge transport across the lithium battery.^8^

On the other hand, the inorganic ceramic solid electrolytes also result in attractive approaches due to the high voltage and thermal stability, compatibility with lithium metal, and fast Li^+^ transport.^9−14^ Several types of solid electrolytes have been investigated and, among them, Li_1+*x*_Ti_2–*x*_Al_*x*_(PO_4_)_3_ (LATP) evidence promising properties such as high ionic conductivity (10^–4^–10^–3^ S·^–1^), superior air stability, high oxidation voltage (∼6 V), and raw materials low prices.^15^ In general, the LATP ionic conductivity is governed by the microstructure, mainly grains and grain boundaries.^16,17^ Hence, higher density and larger grain size are required to decrease the grain boundary impedance, which is achieved at sintering temperatures higher than 900 °C.^18,19^ However, LATP has demonstrated modest electrochemical performance attributed to interfacial incompatibilities with the electrodes.^20,21^ For instance, on the cathode side, apart from the low contact electrode–electrolyte, depletion of Li on the interface is observed due to migration to the cathode during charge.^21^ On the negative side, contact with metallic Li causes Ti^4+^ reduction, generating a chemically unstable interface growth and cycling failure.^22^ Therefore, when these interfacial issues are not solved, LATP cells fail when cycling at room temperature^23^ or, if some capacity can be reached, in a few hours it decays due to the overall resistance increase.^24^

In this context, hybrid electrolytes based on a solid matrix and ILs have emerged as an alternative design strategy to overcome individual electrolyte limitations. Quasi-solid hybrid electrolytes demonstrate high ionic conductivity and good chemical stability.^25−27^ Previous works^28−31^ obtained different hybrid electrolytes by mixing ceramic particles and ionic liquid. So, Kim et al.^28^ reported the Li_7_La_3_Zr_2_O_12_ (LLZO)–1-butyl-1-methylpyrrolidinium bis(trifluoromethylsulfonyl)imide (Pyr_14_TFSI)–lithium bis(trifluoromethanesulfonyl)imide (LiTFSI) hybrid electrolyte, which presented an ionic conductivity of 0.4 × 10^–3^ S·cm^–1^ and an electrochemical stability of 5.5 V vs Li/Li^+^. The authors suggested that the IL reduces the grain boundary resistance of LLZO ceramic particles in hybrid electrolytes, facilitating Li^+^ transport at the solid–liquid interface. Furthermore, for different inorganic matrices,^29,30^ it was evidenced that the ceramics have a strong interaction with many anions of the ionic liquid. Hence, anion trapping by ceramic particles increases the free Li-ion loading, improving Li transport. Additionally, a previous report^31^ evidenced that Li_1.5_Al_0.5_Ge_1.5_(PO_4_)_3_ (LAGP) powder and IL produced a hybrid electrolyte that allowed the creation of a chemically stable electrode/electrolyte interface with low resistance and efficiently avoided thermal runaway at elevated temperatures (575 K). Although, as mentioned above, there are works in the literature using inorganic solid electrolytes and ionic liquids, no hybrid rigid solid electrolytes using porous ceramic LATP have been found.

Following our previous work based on sodium hybrid electrolytes,^32^ we present here a rational design of hybrid lithium electrolytes based on highly porous ceramic in which IL has been infiltrated. The highly porous ceramic matrix with a complex pore network was attained by a simple process employing different corn starch loadings as the pore former. Subsequently, the obtained porous ceramic matrices were filled with 9:1 N-butyl-*N*-methylpyrrolidinium bis(trifluoromethanesulfonyl)imide: lithium bis(trifluoromethanesulfonyl)imide (LiTFSI) (Pyr_14_TFSI–LiTFSI) to achieve a quasi-solid-state hybrid electrolyte and the physicochemical characteristics, hardness, and ionic conductivity were exhaustively analyzed. The designed hybrid electrolytes present elevated ionic conductivity (∼10^–3^ at 303 K), improved Li^+^ transference number, and wide electrochemical window (4.7–4.9 V). Furthermore, it was also evidenced that the LATP support porosity plays a critical role. The porosity increases favor the TFSI^–^-confinement effect at the ceramic interfaces as a result of higher TFSI^–^ and LATP interaction. Hence, free Li^+^ increases influence ionic conductivity and transference number.

## 2 Experimental Section

### 2.1 Preparation of LATP–IL Electrolytes

The hybrid electrolytes were prepared by infiltration of a well-known IL into a highly porous sintered sample of Li_1.3_Al_0.3_Ti_1.7_(PO_4_)_3_ (LATP). So, first, the ceramic porous matrix of LATP was obtained by solid-state sintering of a mixture of LATP powder (Geloon, China) with 5 and 10 vol % of corn starch (CS) as the pore former (samples labeled as LATP-5CS and LATP-10CS). The powders were first mixed in an agate mortar and then ball-milled with acetone for 6 h at 350 rpm. Subsequently, the samples were heated at 333 K for 24 h to remove the solvent, and the final mixtures were pelletized (13 mm diameter and ∼1 mm thickness) by applying 150 MPa uniaxial press. The solid electrolytes’ sintering was performed in a furnace at 1273 K for 24 h using a heating rate of 1 K min^–1^.

**Figure 1:**
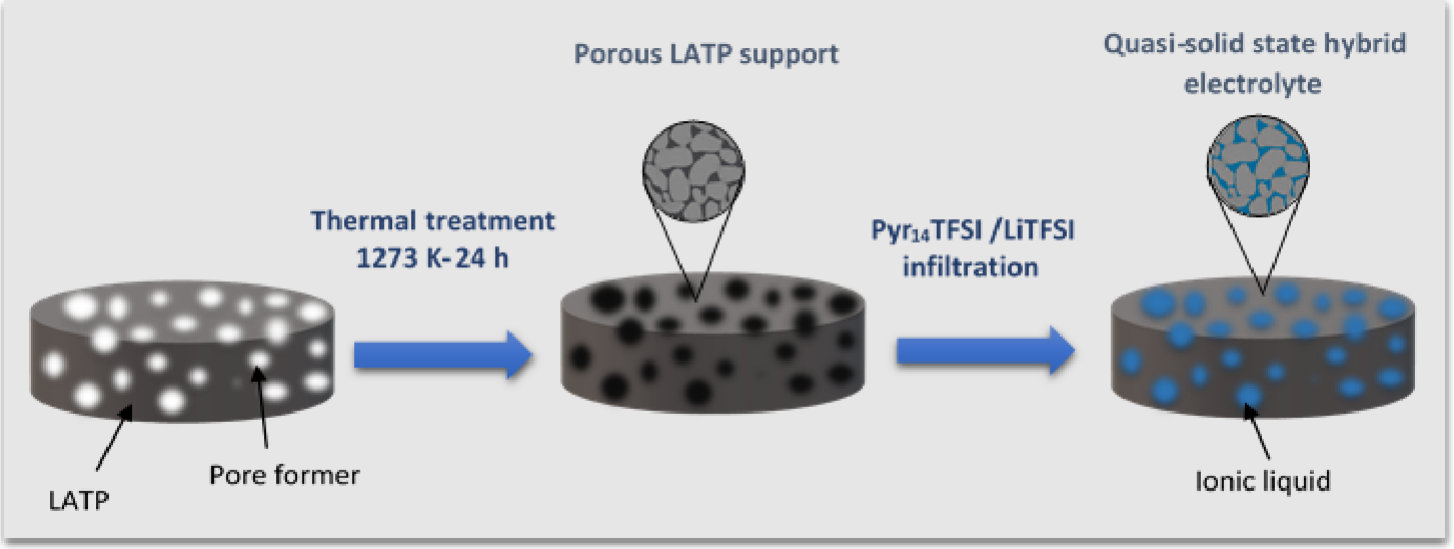
Synthesis process of the LATP–CS–IL hybrid electrolytes.

Afterward, a solution of 1-butyl-1-methylpyrrolidinium bis (trifluoromethanesulfonyl)imide (Pyr_14_TFSI): lithium bis(trifluoromethanesulfonyl)imide (LiTFSI) in 9:1 mol ratio (IL–LiTFSI, 99,9 Solvionic, France) was infiltrated into the porous LATP. The infiltration process was performed in a glovebox (argon atmosphere (O_2_ ≤ 1 ppm; H_2_O ≤ 10 ppm)) by adding the ionic liquid solution to LATP supports and applying a vacuum for 12 h to ensure the complete incorporation into the pores. The synthesis of the hybrid electrolyte process is illustrated in Figure 1.

### 2.2 Characterization Techniques and Electrochemical Studies

Morphological analysis of porous microstructure was performed by using field-emission gun scanning electron microscope (FEG-SEM, FEI TENEO) operating at 10 kV. Moreover, LATP pellets microhardness characterization was carried out by employing Vickers microhardness on Zwick/Roell tester and applying a load of 0.5 kgf during 10 s. At least 20 indentations were performed per sample to attain reproducibility, and the mean value was reported.

The ceramic samples’ density was determined by Archimedes’ principle-based method employing a density kit (balance Sartorius CP225D). To attain the apparent density, the pores were blocked, covering sintered samples with a thick varnish layer. Following this, the covered samples were immersed in deionized water and apparent density was calculated according to
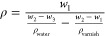
1where *w*_1_: sample weight in the air; *w*_2_: varnished sample weight in the air; *w*_3_: varnished sample weight immersed in water; ρ_water_: deionized water density; ρ_varnish_: varnish density (1.1 g·cm^–3^). Measurements were performed in triplicate. Complementarily, the close porosity was estimated using a pycnometric density measurement of both LATP powder and sintered samples. The pycnometric density was carried out on Micro-Ultrapyc 1200e gas pycnometer (Quantachrome Instruments), equipped with a 0.25 cm^3^ sample holder. For each density measurement, ∼ 100 mg of sample was used.

Additionally, the surface wettability of sintered porous samples with Pyr_14_TFSI–LiTFSI IL was analyzed through a physical contact angle measurement using an OCA 15plus contact angle system (Neurtek Instruments, Spain). The analysis was standardized by employing a 1.5 μL IL drop, which was placed on the pellets’ surface. Raman spectra for LATP–IL samples were measured using B&W Teki-Raman Plus (Newark, USA) spectrometer. Laser radiation was operated at a wavelength of 532 nm, with a spectral resolution of 4 cm^–1^, laser power of 10%, and exposure time of 30 s.

The ionic conductivity was evaluated by impedance spectroscopy employing an Impedance/Gain-Phase Analyzer SI1260 Solartron. AC measurements applying a signal of 100 mV were performed in the frequency range from 0.1 Hz to 1 MHz and temperature range from −243 to 363 K with 10 K intervals. The samples (∼0.8 cm in diameter) were covered with gold paint and acting as blocking electrodes. The complex impedance plot was used to determine the bulk resistance and σ_dc_ was calculated according to the eq 2:
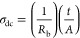
2where *t* and *A* are the thickness and area of the electrolyte, respectively, and *R*_b_ is bulk resistance. The lithium-ion transference number (*t*_*Li*_+) was estimated from the dc/ac method proposed by Bruce et al.^33^ In this method, the initial (*I*_0_) and steady-state (*I*_ss_) currents were measured by applying a constant potential of 5 mV through a symmetrical Li|electrolyte|Li cell. At the same time, the cell was monitored by impedance spectroscopy to determine the initial (*R*_0_) and steady-state resistances (*R*_ss_) of the two Li interfaces. The *t*_*Li*_+ was calculated according to
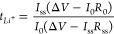
3

The electrochemical stability window of the obtained electrolyte was evaluated in a lithium/electrolyte/stainless steel cell configuration by the linear sweep voltammetry (LSV) method at 0.1 mV·s^–1^ from 2 to 5 V vs Li^+^/Li. Moreover, the electrochemical performance was evaluated by galvanostatic charge–discharge (GCD) tests using a two-electrode coin cell (CR2032) configuration with a Neware BTS-4000 battery tester. The electrode thickness was reduced to 500 μm for galvanostatic tests. A metallic Li disk (Gelon LIB co., China) was used as the negative electrode in excess with respect to the positive electrode. For the positive electrode, a slurry containing LiFePO_4_ (LFP) (1.7% C-coated, Gelon LIB Co., China) – Super C65 (C-nergy, France)-PVDF (Gelon LIB Co., China) in a weight ratio of 80:10:10 was mixed overnight in an NMP solution. The slurry was cast on Al foil with a Doctor Blade device, dried at 80 °C and punched into 8 mm diameter electrodes with a mass loading of 6 mg·cm^–2^. To develop the full cell, Li_4_Ti_5_O_12_ (LTO) (Gelon LIB Co., China) electrodes were prepared with the same method and the same proportions of additives resulting in discs of 8 mm diameter and 10 mg cm^–2^. Cell impedance measurements were taken in the discharged state after 1 h at rest at room temperature using the same equipment as for the electrolyte conductivity measurements. C-rates were calculated based on LFP theoretical capacity (170 mAh·g^–1^). Lithium stripping-plating tests were carried out using symmetric cells with Li as the working and counter electrode. Positive and negative 0.1 mA cm^–2^ currents were alternatively set for 1 h each with limiting voltages of 1 and −1 V.

## 3 Results and Discussion

### 3.1 Sintered LATP Characterization

**Figure 2:**
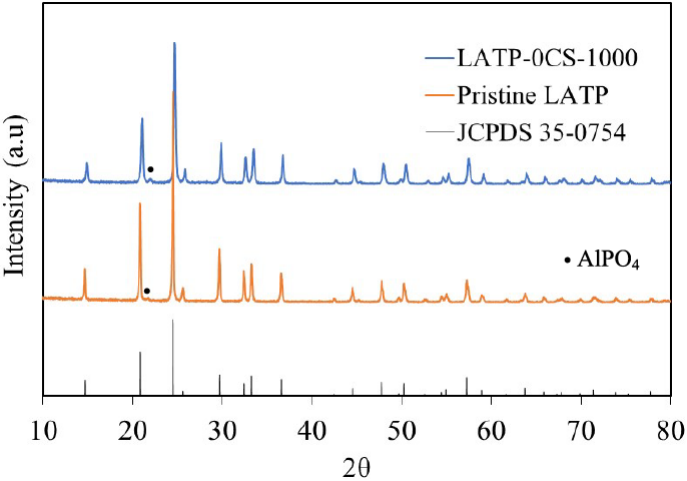
XRD patterns of pristine LATP and LATP-0CS sintered at 1000 °C.

The powder X-ray diffraction patterns of LATP pristine powder and pellet synthesized at 1000 °C are presented in Figure 2. All of the diffraction peaks are in good agreement with NASICON-type LATP (JCPDS 35–0754). Furthermore, the small intensity peak observed in the diffractogram of pristine LATP powder, and assigned to AlPO_4_, does not undergo an increase in intensity in the sintered samples. Moreover, the corresponding elemental mapping (Figure S1) of the LATP-0CS sample shows a homogeneous distribution of P, Al, and Ti elements after the sintering process at 1000 °C.

**Figure 3:**
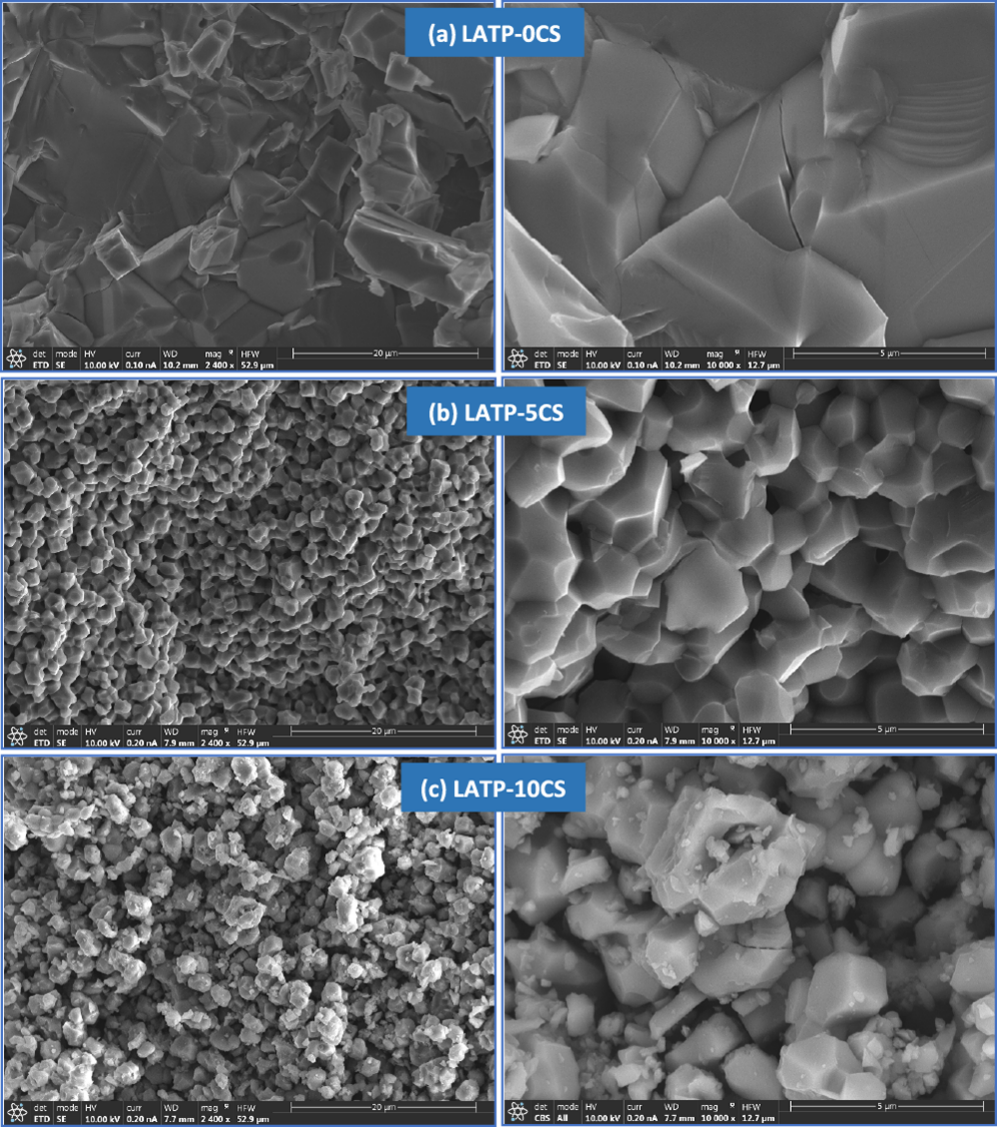
SEM micrographs of LATPS-0CS (a), LATP-5CS (b), and LATP-10CS (c).

To evaluate the suitable manufacture of highly porous ceramic supports for hybrid electrolyte purposes, two volumetric concentrations (5% and 10%) of the pore former were employed. Figure 3 shows the SEM micrographs of sintered LATP-5CS and LATP-10CS samples. The resulting microstructures are compared with LATP-0CS (without a pore former). From Figure 3a it is observed that LATP-0CS pellets present a typical microstructure of dense ceramic, with equiaxial grains and few pores, while LATP-5CS (Figure 3b) and LATP-10CS (Figure 3c) samples exhibit as expected a higher porosity, derived from corn starch pore-former pyrolysis, and less sintering. Also, in the LATP-0CS sample is observed the intragrain fracture of some crystals, a characteristic of the well-sintered ceramic samples, while the porous LATP-5CS and LATP-10CS display an intergrain fracture. Thus, porous samples present small particles and well-distributed microporosity (<2 μm). Additionally, as shown in Figure 3, the samples show different degrees of sintering depending on the % corn-starch. These differences are explained by the lower degree of consolidation (or green density) of the sample just after elimination of the pore former. Thus, the LATPS-10CS sample with a lower density just before the onset of the sintering process shows a lower degree of consolidation showing small visible pristine particles (less than 1 μm) that are not incorporated into the sintered monolith. In contrast, the LATP-5CS porous support shows no pristine unincorporated particles, indicating a more sintered structure. Finally, the sample without the pore former presents the highest degree of sintering, where, unlike the previous samples, grain growth has occurred.

**Figure 4:**
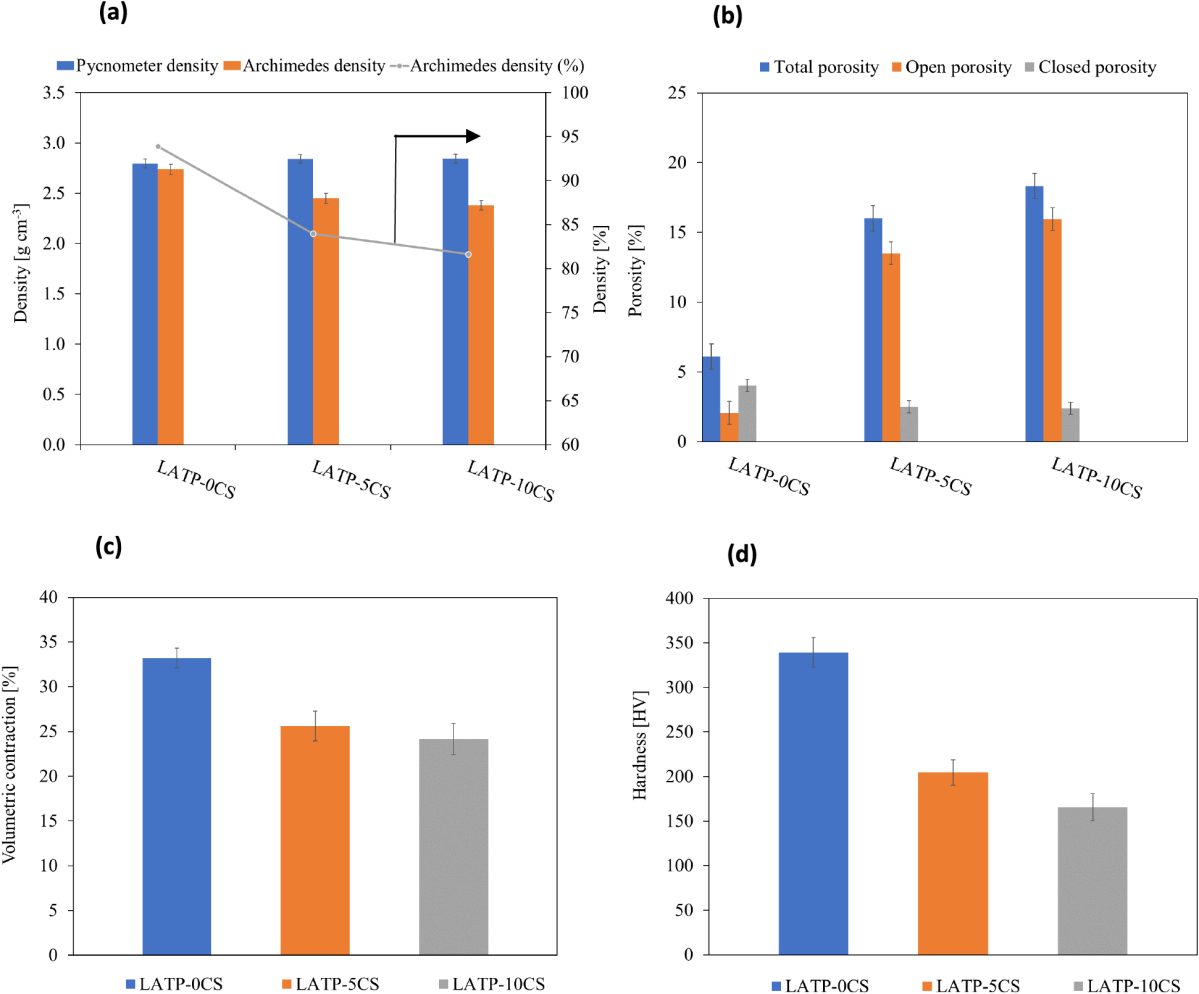
Pycnometer and Archimedes density (a), porous content (b), ceramic matrix volumetric concentration (c), and hardness (d).

Furthermore, the ceramic pellets’ densification and porosity concentration were analyzed by a combination of helium pycnometer measurements and Archimedes’ principle-based method. Helium pycnometer gives the closest approximation to the true density since helium penetrates the smallest pores and crevices approaching the real volume. The results of pycnometer density involving ceramic bulk and closed pore volumes show no significant differences between the LATP pellet without a pore former and LATP pellets with different pore-former loadings (Figure 4a). As expected, apparent density (Archimedes density) including ceramic bulk, and open, and closed pore volumes diminish with the pore-former loading increase because open porosity increases. In addition, as seen in Figure 4b the closed porosity (isolated pores) concentration is low; i.e., 4% for LATP-0CS, and decreases to 2.5% and 2.4% for LATP-5CS and LATP-10CS, respectively. Conversely, the open porosity (interconnected pores) increases from 2.4% (LATP-0CS) to 13.5% and 15.9% for LATP-5CS and LATP-10CS, respectively. These results are in good agreement with the observed microstructure, where higher porosity and more disconnected particles correspond to an increase in the pore-former loading.

The postsintering volumetric contraction (Figure 4c) for LATP-0CS is approximately 33.2%, while for LATP-10CS and LATP-5CS it is slightly lower, being 25.6% and 24.2%, respectively. Complementarily, mechanical properties were analyzed from Vickers microhardness measurements. To obtain reproducible values, 20 repetitions were taken at different points of the sample surface. The obtained results (Figure 3d) show a decrease in the hardness of the samples with a pore former compared to those without a pore former. These results are in good agreement with the observed porosity of sintered samples, where higher porosity corresponds to microhardness decrease.

In general, during the sintering process, the pyrolyzable pore-former agent undergoes decomposition, creating well-defined pores. Thus, the resultant porous LATP matrices with 14–16% of interconnected channels result in an appropriate 3D structured matrix that could contribute further to alternative ion transport pathways in hybrid electrolytes.

**Figure 5:**
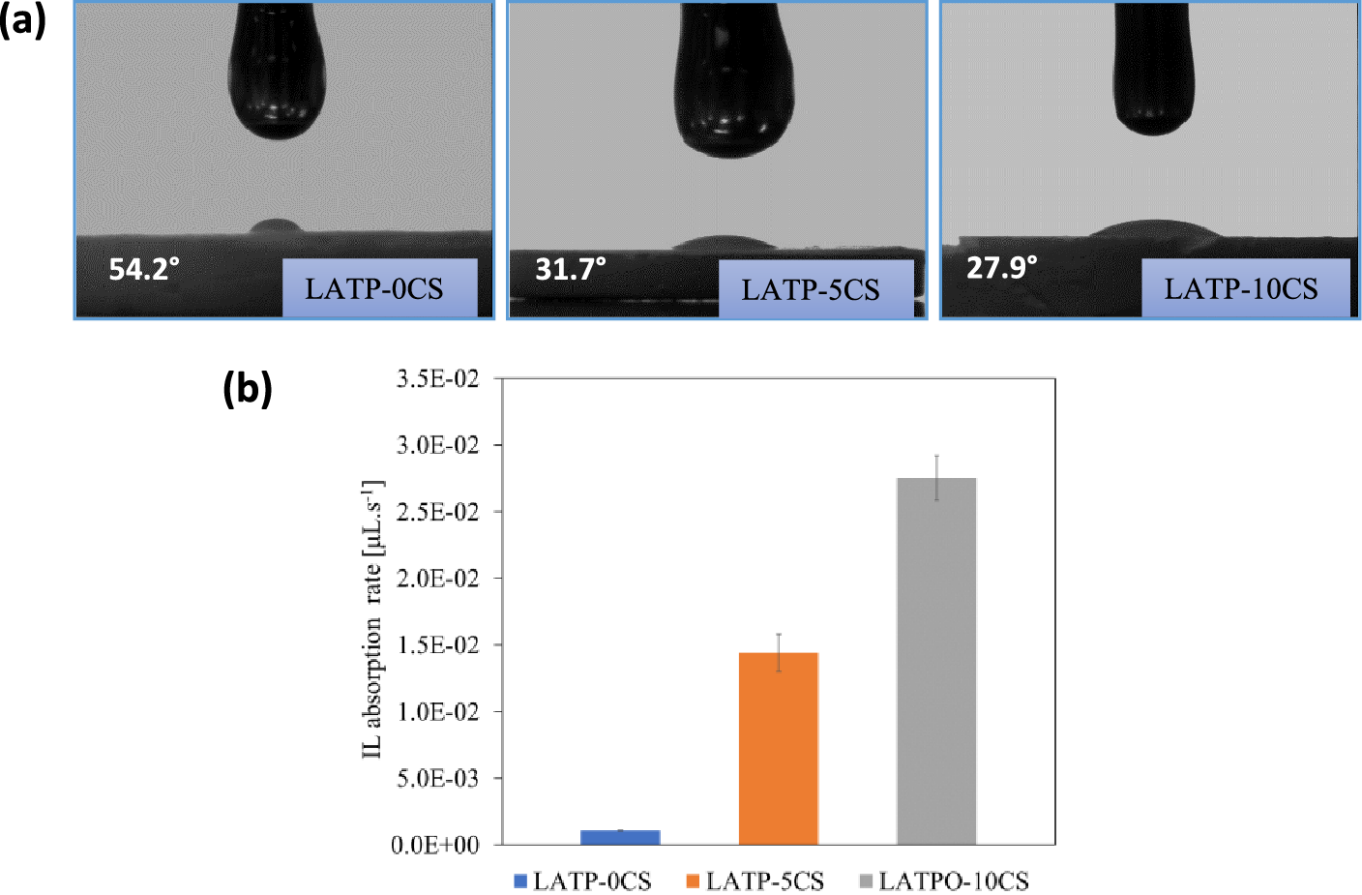
Images of contact angle (a) and absorption rate (b) of the IL–LiTFSI drop after deposition on LATP-0CS, LATP-5CS, and LATP-10CS surfaces.

Once the porous ceramics were obtained, the quasi-solid hybrid electrolytes were manufactured by infiltration of Pyr_14_TFSI–LiTFSI solution into the LATP-0CS, LATP-5CS, and LATP-10CS matrices, and the interactions between the porous matrix and IL-LiTFSI filler were further analyzed. First, the Pyr_14_TFSI–LiTFSI infiltration capacity within porous ceramics was evaluated from the surface wettability test. Figure 5a shows the images of IL–LiTFSI drops after 10 s deposition on the LATP surfaces with different porosities. As expected, the results display that the filler droplet on the LATP-0CS sample creates a high contact angle, while the porous LATP-5CS and LATP-10CS matrices show low contact angles, which demonstrates that, as expected, the porous morphology of ceramic electrolytes improves the IL–LiTFSI wettability. Complementarily, the rate of Pyr_14_TFSI–LiTFSI absorption was performed from the measurement of the required time for complete absorption of IL-LiTFSI specific volume (1 μL). The obtained results (Figure 5b) indicate that the IL adsorption rate increases according to the following sequence LATP-10CS > LATP-5CS > LATP-0CS, thus validating that as the ceramic porosity increases, the absorption rate increases.

Accordingly, the LATP-5CS and LATP-10CS microporous structures and their high adsorption capability allow their applications as promising reservoirs for liquid electrolytes, which are retained in the rigid matrix by capillary effects.

### 3.2 Electrical and Electrochemical Characterization

The ionic conductivity of the porous ceramic matrices and quasi-solid hybrid electrolytes obtained by AC impedance measurements was between 243 and 363 K. First, the temperature dependence of the ionic conductivity of the LATP ceramic supports is shown in Figure S2. As expected, the conductivity of the studied systems increases with the temperature and fulfills the Arrhenius-type dependence. The determined values of total conductivity at 303 K, activation energy, and pre-exponential factor are listed in Table S1. The obtained results for LATP-0CS sintered at 1273 K are comparable with previous reports.,^34−36^ In addition, it was observed that the total ionic conductivity strongly depends on the microstructural features (density, porosity morphology, and particle size). Accordingly, LATP-0CS with a dense structure and lower porosity showed the lowest activation energy and highest conductivities over the entire temperature range regarding the porous LATP-5CS and LATP-10CS support.

**Figure 6:**
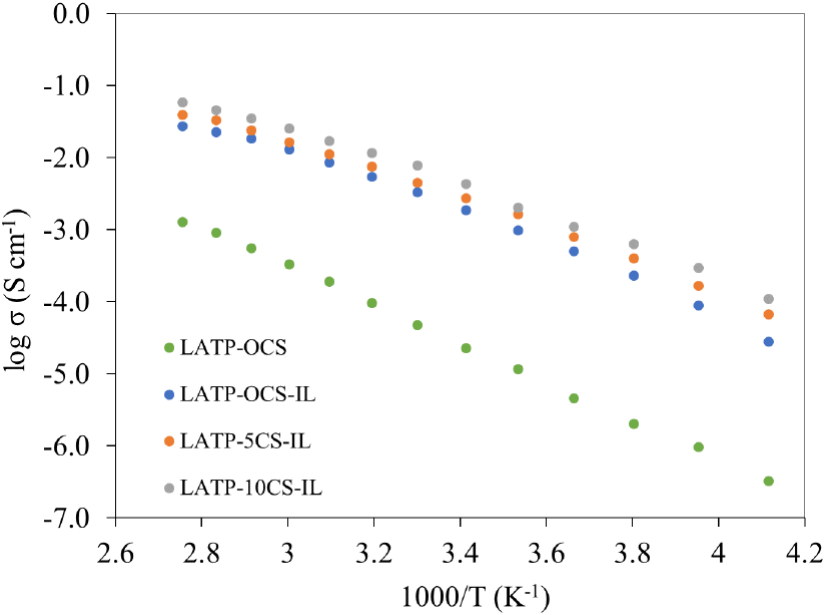
Arrhenius plots for LATP supports and quasi-solid-state hybrid electrolytes.

Furthermore, for the proposed quasi-solid-state hybrid electrolytes, the obtained results evidence a significant improvement in two–three magnitude orders of the total ionic conductivity regarding ceramic supports. The temperature dependence on the conductivity of the hybrid systems is shown in Figure 6. It is observed that the total ionic conductivities of the hybrid systems are above 10^–4^ S·cm^–1^ at 263 K and increase to ∼10^–2^ S·cm^–1^ at 363 K. Moreover, for comparative purposes, an approximation of the ionic conductivity temperature dependence for Pyr_14_TFSI–LiTFSI IL (Figure S3) was obtained using Glass Microfiber filler (Whatman GF/D) support and its resultant ionic conductivities were comparable with the reported conductivity for pure Pyr_14_TFSI–LiTFSI IL.^37^ In addition, ionic conductivities of the LATP–IL hybrid electrolyte systems are smaller than Pyr_14_TFSI–LiTFSI–GF/D suggesting the existence of chemical interaction between the infiltrated IL and the porous LATP support. Likewise, the higher conductivity of LATP-10CS–IL in comparison to LATP-0CS–IL and LATP-5CS–IL samples could be attributed to the higher porosity of ceramic support that allows the effective solvation of more Li ions with lesser confinement effect.^38^

Additionally, it was evidenced that ionic conductivity increases with temperature rise following Vogel–Tammann–Fulcher (VTF, eq 4) behavior as expected for systems in which the conductivity is essentially governed by IL viscosity^39^:
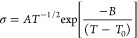
4where *A* is the pre-exponential factor associated with the carrier ion (Pyr_14_^+^, TFSI^–^, and Li^+^) concentration, *B* is the parameter related to the apparent activation energy which corresponds to the required energy to overcome association and become free conductive ions, and *T*_0_ is the temperature at which the configurational entropy becomes zero. The VTF fitting for the hybrid systems is presented in Figure S4 and VTF parameters are listed in Table 1. The resulting values of apparent ion transport activation energy for hybrid conductors are higher than the pristine IL. Thus, a lower value indicates that smaller energy is required for ion migration in GF/D–IL (Table S2), while the higher energy activation for the designed hybrid electrolytes suggests a synergic effect between porous LATP supports and Pyr_14_TFSI–LiTFSI IL, which can be associated with the change in ion conductive path owing to the ordered structure support.

**Table 1:** Overall Electrical Conductivity at 303 and 363 K and Parameters from VTF Equations for Quasi-Solid-State Hybrid Electrolytes

sample	A [S cm^–1^ K^–1/2^]	B [K]	T_0_ [K]	*E*_a_ [eV]	σ [S·cm^–1^] 303 K	σ [S·cm^–1^] 363 K
LATP-0CS–IL	1.38	595	134	0.051	3.4 × 10^–3^	2.7 × 10^–2^
LATP-5CS–IL	1.67	759	113	0.065	4.5 × 10^–3^	3.9 × 10^–2^
LATP-10CS–IL	1.99	820	105	0.071	7.9 × 10^–3^	5.9 × 10^–2^

In this regard, Kwatek et al.^25^ proposed that the improvement in grain boundary electric properties of LATP–IL and LTP–IL composites could be ascribed to (i) the interaction of the adjoining phases or (ii) IL acting as a connector facilitating lithium-ion transport. In addition, Paolella et al.^40^ indicated that both NASICON LAGP and Pyr_13_TFSI–LiTFSI participate in Li ions diffusion by the ionic bridge formation between the two components. Contrarily, Choi et al.^30^ showed that LiTFSI/Pyr_14_TFSI–BaTiO_3_ electrolyte with ionic conductivity of 1.3 × 10^–3^ S×cm^–1^ at 303 K and remarkably high lithium-ion transference number (0.35) presents the formation of positive-charge space around the nanoparticles due to anion trapping by the nanosized BaTiO_3_ particles. Similarly, Kim et al.^29^ indicated that the conductivity difference between Pyr_14_TFSI–LiTFSI and Pyr_14_TFSI–LiTFSI–TiO_2_ is attributable to the reduction in Py_14_^+^ and TFSI^–^ contribution, while free Li-ion contribution increases the ionic conductivity.

**Figure 7:**
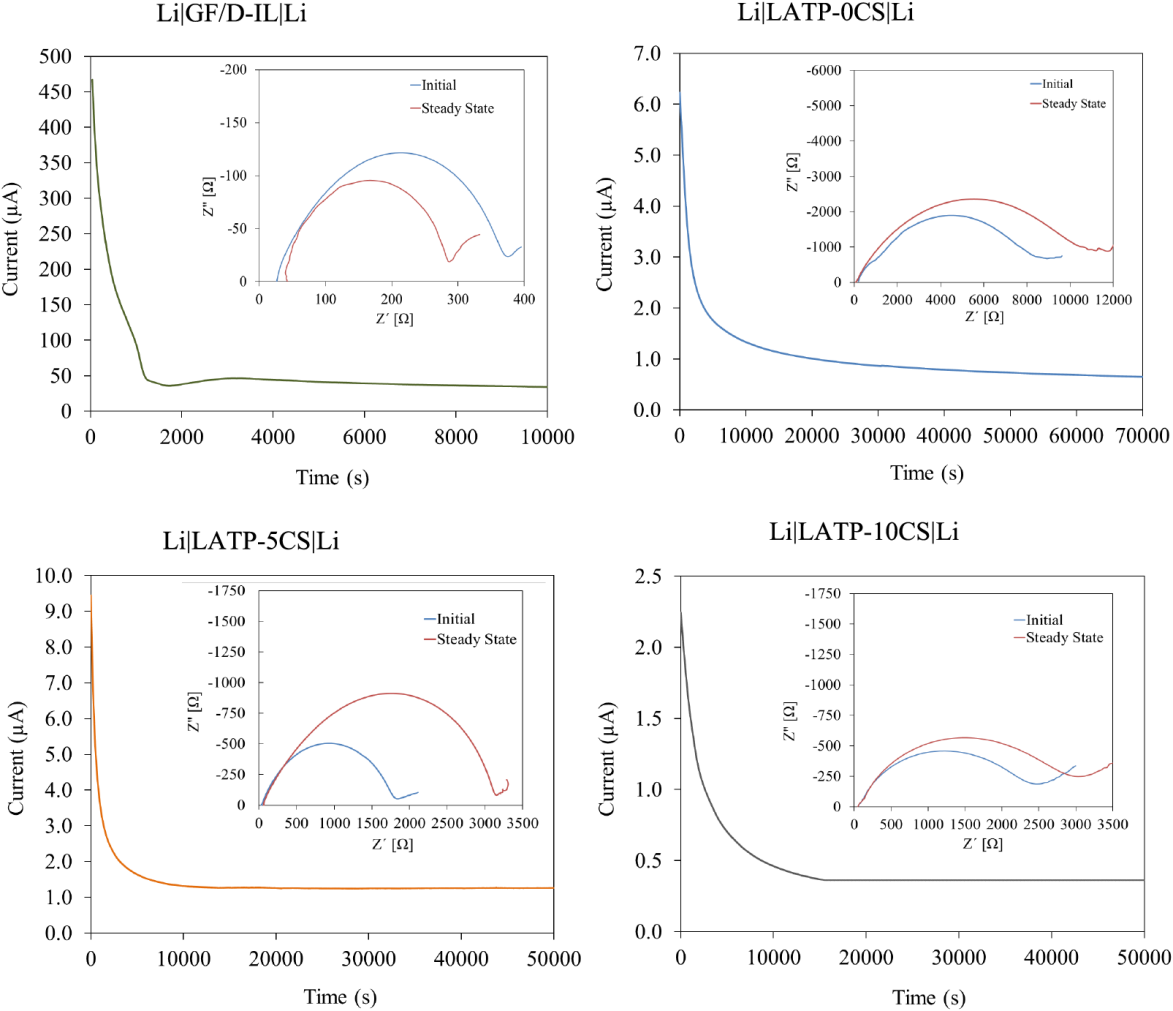
Polarization curve and EIS plot before and after polarization (inset) of GF/D–IL and LATP–IL hybrid electrolytes.

Additionally, the Li-ion dynamics inside the LATP micropores was characterized from *t*_*Li*_+. Higher *t*_*Li*_+ is required to reduce concentration polarization during the charge/discharge process, and consequently, it can avoid Li salt decomposition and precipitation, hinder Li dendrite growth, and increase power density. The *t*_*Li*_+was measured via the steady-state current method proposed by Bruce and Vincent.^26^ The polarization current curves (*I*_Ohm_ and *I*_ss_) and impedance spectra (*R*_i,0_ and *R*_i,ss_) for the Li|electrolyte|Li symmetric cells are shown in Figure 7. The calculated *t*_*Li*_+values of Li|LATP-0CS–IL|Li, Li|LATP-5CS–IL|Li and Li|LATP-10CS–IL|Li were 0.10, 0.13, and 0.18, respectively, which evidence a significant improvement compared with GF/D–IL commercial separators (0.06). This enhancement of practical Li-ion ionic conductivity for the quasi-solid-state conductors could be attributable to the formation of positive-charge space. More specifically, the TFSI^–^ anion could interact with ceramic groups promoting the Li^+^ release and hybrid electrolyte *t*_*Li*_+ increases.

**Figure 8:**
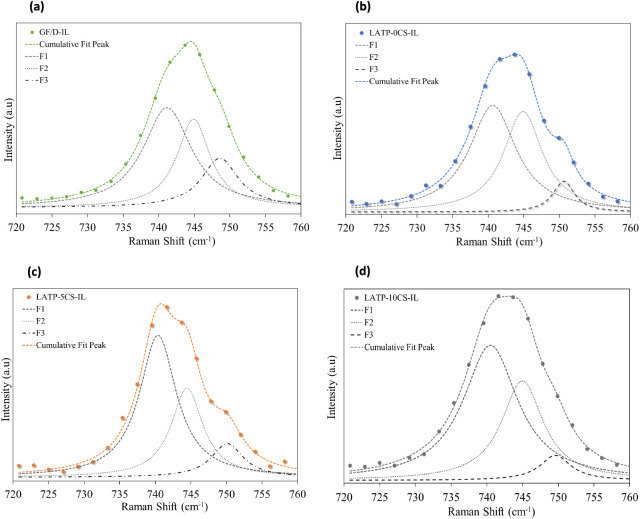
Raman spectra of GF/D–IL (a), LATP-0CS–IL (b), LATP-5CS–IL (c), and LATP-10CS–IL (d) hybrid electrolytes.

Besides, Raman spectroscopy was employed to characterize the molecular features of hybrid electrolytes with high ionic conductivity. Raman mode around 742 cm^–1^ corresponds to the expansion and contraction modes of the TFSI^–^ anion fully solvated by weakly interacting cations. This signal involves two contributions at 738–740 (F1) and 742–745 (F2) cm^–1^ corresponding to C1 (cisoid) and C2 (transoid) conformational states of free TFSI^–^ in equilibrium, respectively. In addition, the third component at 746–750 (F3) cm^–1^ corresponds to TFSI anion interaction with Li^+^ to form ion pairs and aggregates (coordinated to more than one Li^+^ cation).^41−43^ The approximation composition analysis was calculated from the integrated peak areas (Figure 7):
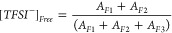
5where *A*_F1_, *A*_F2_, and *A*_F3_ are the integrated peak areas of the fitted peaks F1, F2, and F3, respectively. Figure 8 presents a comparison of Raman spectra between 720 and 760 cm^–1^ of GF/D–IL (a) and LATP-0CS–IL–LiTFSI (b), LATP-5CS–IL–LiTFSI (c), and LATP-10CS–IL–LiTFSI (d) samples. The dot lines in the peak are the results of band fit using Lorentzian profiles. The electrolyte samples present an asymmetric broadening of the signal around 742 cm^–1^, and in fact, the fitted band revealed three bands at ∼740, 745, and 750 cm^–1^. Besides, the free TFSI^–^ concentration was ∼70% for GF/D–IL and when IL was incorporated into the porous LATP support, the free TFSI^–^ anion concentration increased to 93%, 94%, and 88% for LATP-0CS–IL, LATP-5CS–IL, and LATP-10CS–IL, respectively. This result indicates an interaction between TFSI^–^ and the ceramic, which increases the free Li^+^ amount and consequently influences the resultant *t*_*Li*_+ and ionic conductivity. Moreover, it was observed that TFSI^–^ confinement effect at the ceramic interfaces is favored by support porosity increases as a consequence of the higher concentration of available superficial sites for the interaction. Thus, the obtained results are according to previous reports for SiO_2_,^44^ TiO_2_,^22^ and BaTO_3_^23^ supports.

**Figure 9:**
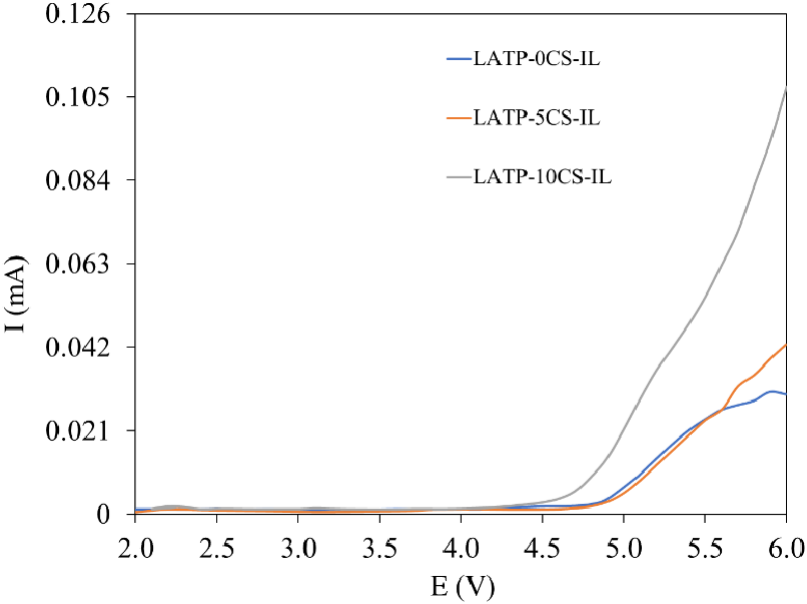
Linear sweep voltammetry of hybrid electrolytes at 0.2 mV s^–1^ with a cutoff limit of 6 V.

The electrolyte electrochemical stability is an essential parameter for Li battery safety. Accordingly, the linear sweep voltammetry (LSV) profiles were obtained for Li/LATP-0CS–IL/SS, Li/LATP-5CS–IL/SS, and Li/LATP-10CS–IL/SS cells at a scan rate of 0.2 mV·s^–1^ in the 2.0–6.0 V range (Figure 9). The obtained results showed good anodic stability with an electrochemical stability window of 4.7 vs Li^+^/Li for LATP-10CS–IL and 4.9 vs Li/Li^+^ for LATP-0CS–IL and LATP-5CS–IL electrolytes. These values are acceptable for use with high-voltage intercalation-compound cathodes employed for lithium batteries, such as LiFePO_4_ and LiCoO_2_. In addition, the obtained LSV voltammograms of hybrid electrolyte cells show that the anodic current does not increase up to 4.7–4.9 V, evidencing the absence of any other electrochemical reactions in the cells until their electrochemical windows.

**Figure 10:**
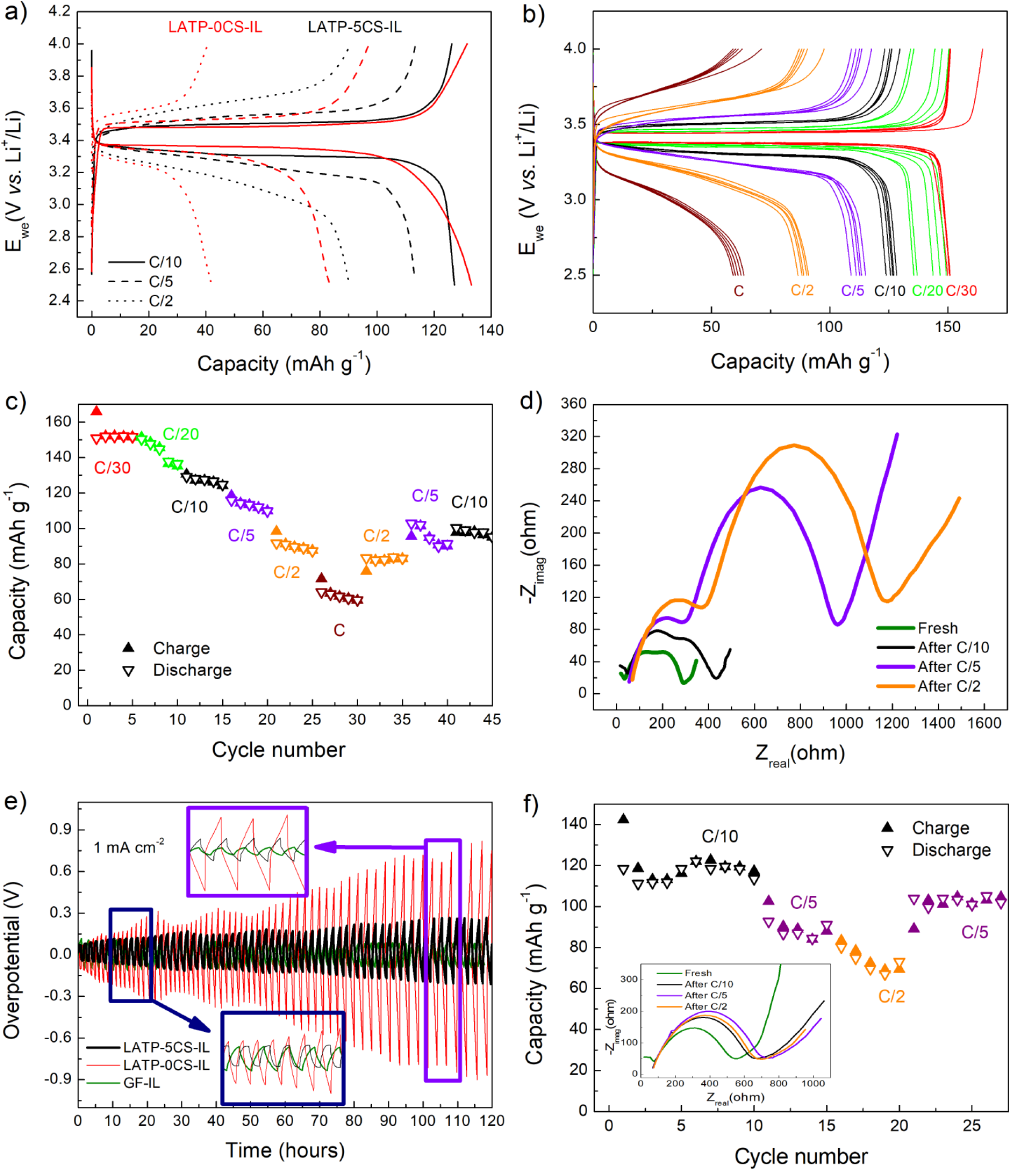
Comparison of voltage profiles of LATP-0CS and LATP-5CS in LFP half cells at C/10, C/5, and C/2 (a), voltage profiles (b), and capacity vs cycle number (c) at different C-rates (C/30, C/20, C/10, C/5, C/2, and 1C) for a LFP/LATP-5CS–IL/Li cell. EIS Nyquist diagrams of a fresh LFP/LATP-5CS–IL cell, after 5 cycles at C/10, then other 5 cycles at C/5 and 10 cycles at C/2 (d). Li stripping and plating tests for LATP-0CS–IL, LATP-5CS–IL, and glass fibers with IL at 0.1 mA·cm^–2^ (e). Capacity vs cycle number for a full cell LFP/LATP-5CS–IL/LTO at C/10, C/5, and C/2 (f) and EIS Nyquist diagrams of the fresh cell and after each change of rate (inset of f). The electrochemical experiments have been carried out at room temperature (20 °C).

The electrochemical performance of LATP-0CS–IL and LATP-5CS–IL electrolytes (500-μm thickness) was tested at room temperature (20 °C) in CR2032 cells using LFP and Li as positive and negative electrodes, respectively. First, cells using LATP-10CS as the SE were assembled, but the higher porosity confers low mechanical stability. Figure 10a shows a comparison of the voltage profile obtained at C/10, C/5, and C/2 for each electrolyte. At C/10 similar capacities are achieved for both electrolytes. However, at lower rates (C/5 and C/2), a lesser performance for LATP-0CS–IL was detected. A slight voltage drop can be identified at the end of the discharge at C/5, which can originate from the higher concentration polarization that may be attributable to the lower amount of ions rapidly available on LATP-0CS–IL. Figure 10b,c shows the results of a rate capability test using LATP-5CS–IL. The cell delivered an initial reversible capacity of 150 mAh g^–1^ LFP at C/30. An increase in the overpotential of the charge and discharge curves can be observed during cycling and may be related to the capacity fading. While the IL is expected to be stable in LFP half-cells,^45^ LATP is known to react with Li causing spontaneous reduction of Ti^4+^ and degradation of lithium/electrolyte interface leading to an increase in the impedance with time.^46−48^Figure S5 shows the pictures of LATP-5CS and LATP-5CS–IL pieces and the IL-impregnated glass fiber separator that were in contact with lithium foil for 24 h. Color changes can be observed on the LATP-5CS and LATP-5CS–IL samples but not in the IL-impregnated separator. The total impedance increase is confirmed in the Nyquist plots (Figure 10d) which reveals the variation after 5 cycles at C/10, 5 cycles at C/5, and 10 cycles at C/2. The changes are more significant after operating at C/5 than after operating at C/10, indicating that higher rates lead to greater degradation. Likewise, the performance of Li/electrolyte/Li symmetric cells in Li stripping and plating tests (Figure 10e) corroborates the degradation of the Li/electrolyte interface with a constant increment of the overpotential upon cycling for LATP-5CS5–IL and LATP-0CS–IL, while it was stable for IL. It is worth mentioning that LATP-5CS–IL displays a much lower overpotential than LATP-CS10.

Despite the fading and the interface degradation, the cell still releases 60 mAh·g^–1^ LFP at 1C. It should be noted that these results were obtained at 20 °C. Previous works in the literature using LATP did not show satisfactory results at room temperature, even when using much thinner electrodes. Yang et al. with LFP electrodes of 0.02 mg· cm^–2^, bare LATP, and Li foil only obtained 20 mAh·g^–1^ at 0.1 C at room temperature.^23^ Better results in terms of capacity were shown by Yang et al.^24^ with electrodes of 1 mg·cm^–2^ but adding some liquid organic electrolyte on the surface of LATP pellet to facilitate contact with the electrode. 150 mAh·g^–1^ were obtained at 1.5 C but the capacity rapidly fell after 100 cycles to zero (<48 h) due to the resistance increase.

After slowing the rate again to C/10 (Figure 10c), 100 mAh g^–1^ were recovered vs the 125 mAh·g^–1^ obtained initially for the same rate. Nevertheless, this hybrid electrolyte retained these values after 1 month of operation. Compared to a hybrid system containing LATP–PVDF–TrFE–IL,^49^ in that case, 45 mAh·g^–1^ were obtained at 1 C for NMC 811, which has a higher theoretical capacity than LFP used in this work. Regarding the long cycling, the reported NMC811/Li cell kept at 80% of the capacity after 112 cycles at 0.5 C (∼6 days).

To prove that the main limitation in terms of stability comes from the use of lithium metal, a full cell with LTO as a negative electrode has been characterized (Figure 10f). 120 mAh·g^–1^ are obtained at C/10 and 80 mAh·g^–1^ at C/2. Most notably, the total cell resistance does not increase with cycling (see the inset of Figure 10f). The Nyquist diagrams do not vary significantly after each of the different rates. Furthermore, the cell can recover the values obtained when cycled back to C/5 unlike what happens in the half cell.

Although the cycling stability of LATP vs Li can be improved with interfacial optimization techniques, the high capacities obtained at room temperature prove the excellent Li-ion mobility of the hybrid electrolyte proposed here. The improvement of LATP-CS5–IL with respect to LATP-CS0–IL makes the use of corn starch a simple and attractive strategy to improve the performance of IL–ceramic hybrid electrolytes.

## 4 Conclusion

In this work, hybrid electrolytes composed of a porous LATP ceramic and Pyr_14_TFSI–LiTFSI (9:1) ionic liquid are proposed. The LATP ceramic supports with different porosities were obtained by sintering LATP powders with different amounts of corn starch (5 and 10 vol %) which acts as a pore former. Subsequently, the quasi-solid-state hybrid electrolytes were prepared by infiltration of the ionic liquid solution into the ion-conducting porous ceramic. The hybrid electrolytes show an enhanced ionic conductivity with respect to the dense LATP (around 10^–3^ S·cm^–1^ at 303 K, which increases up to one magnitude order (∼10^–2^ S·cm^–1^) at 363 K). Moreover, the Li-ion transference number was improved from pristine ionic liquid from 0.06 to 0.16 by the synergetic effect between the ionic liquid and Li-ion conductor support. The quasi-solid electrolytes present wide electrochemical stability of 4.7–4.9 V. Thus, the incorporation of a porous LATP support allows TFSI^–^ anion interaction with ceramic groups promoting the Li^+^ release and enhancing the ion transference number. In addition, it was observed that this TFSI^–^ confinement effect is favored by support porosity increases, which allows more Li^+^ effective solvation. Finally, the electrochemical performance of the hybrid electrolyte using LiFePO_4_ as a positive electrode and Li metal as a negative electrode was evaluated at room temperature. Despite the considerable thickness of the tested electrolyte and the unoptimized LATP–Li interface, a high capacity of 150 mAh·g^–1^_LFP_ at C/30 was successfully achieved at room temperature, while retaining 60 mAh g^–1^_LFP_ at 1 C. Moreover, 80% of the capacity is kept after the rate capability and one-month total operation time demonstrating better stability than previous works using LATP or LATP–polymer–IL hybrid as SE vs Li before any interface modification. The use of corn starch as a pore generator, previous to infiltration of IL into LATP have been proved to significantly enhance the electrochemical performance of LATP–IL hybrid electrolyte, displaying higher capacities at high rates and lower polarization toward lithium stripping and plating than the nonmodified electrolyte. The development of a full cell with good electrochemical performance in terms of capacity and showing no significant signs of degradation after cycling at C/2 demonstrates that LATP-CS5-IL can be a promising option for Li-metal batteries if interfacial enhancement techniques are applied. These findings establish the hybrid electrolyte as a highly viable substitute for the current organic electrolytes in the next generation of batteries.

## Supporting Information Available

EDS mapping corresponding to the O, Al, P, and Ti distribution after sintering; Arrhenius plots for LATP supports with different microstructures before IL infiltration; table summarizing the electrical conductivity at 303 and 363 K, activation energy (*E*_a_) and the pre-exponential term of Arrhenius expression for the three ceramic LATP supports; Arrhenius plots for Pyr_14_TFSI–LiTFSI IL; table with overall electrical conductivity at 303 and 363 K and parameters from VTF equations for quasi-solid-state hybrid electrolytes; VTF fitting for glass fiber containing IL and the three ceramic supports infiltrated with IL; pictures showing the visual changes of glass fiber impregnated with IL, a piece of LATP-5CS and a piece of LATP-5CS–IL after having been in contact with metallic lithium for 24 h (PDF)
