# Supplementary material for: Advancements in Quasi-Solid-State Li Batteries: A Rigid Hybrid Electrolyte Using LATP Porous Ceramic Membrane and Infiltrated Ionic Liquid
Source: ACS Appl Energy Mater. 2024 Feb 6;7(4):1527–38. doi: 10.1021/acsaem.3c02828 (PMC10900572; doi:10.1021/acsaem.3c02828)
Supplement: Supplementary file 1 — ae3c02828_si_001.pdf [file ae3c02828_si_001.pdf]

Supporting Information

Advancements in Quasi-Solid-State Li Batteries: A Rigid Hybrid Electrolyte Using LATP Porous Ceramic Membrane and Infiltrated Ionic Liquid

Deborath M. Reinoso[†, ‡,*] *Carmen de la Torre-Gamarra*[†], *Antonio J. Fernández-Ropero*[†], *Belén Levenfeld*[†], *Alejandro Várez*[†,*]

[†] Departamento de Ciencia e Ingeniería de Materiales e Ingeniería Química, Universidad Carlos III de Madrid, Avda. Universidad 30, 28911, Leganés, Spain.

[‡] Instituto de Química del Sur (INQUISUR), CONICET, Departamento de Química, Universidad Nacional del Sur (UNS), Avda. Alem 1253, 8000, Bahía Blanca, Argentina.

*Corresponding authors: deborath.reinoso@uns.edu.ar (D. Reinoso), alvar@ing.uc3m.es (A. Várez)

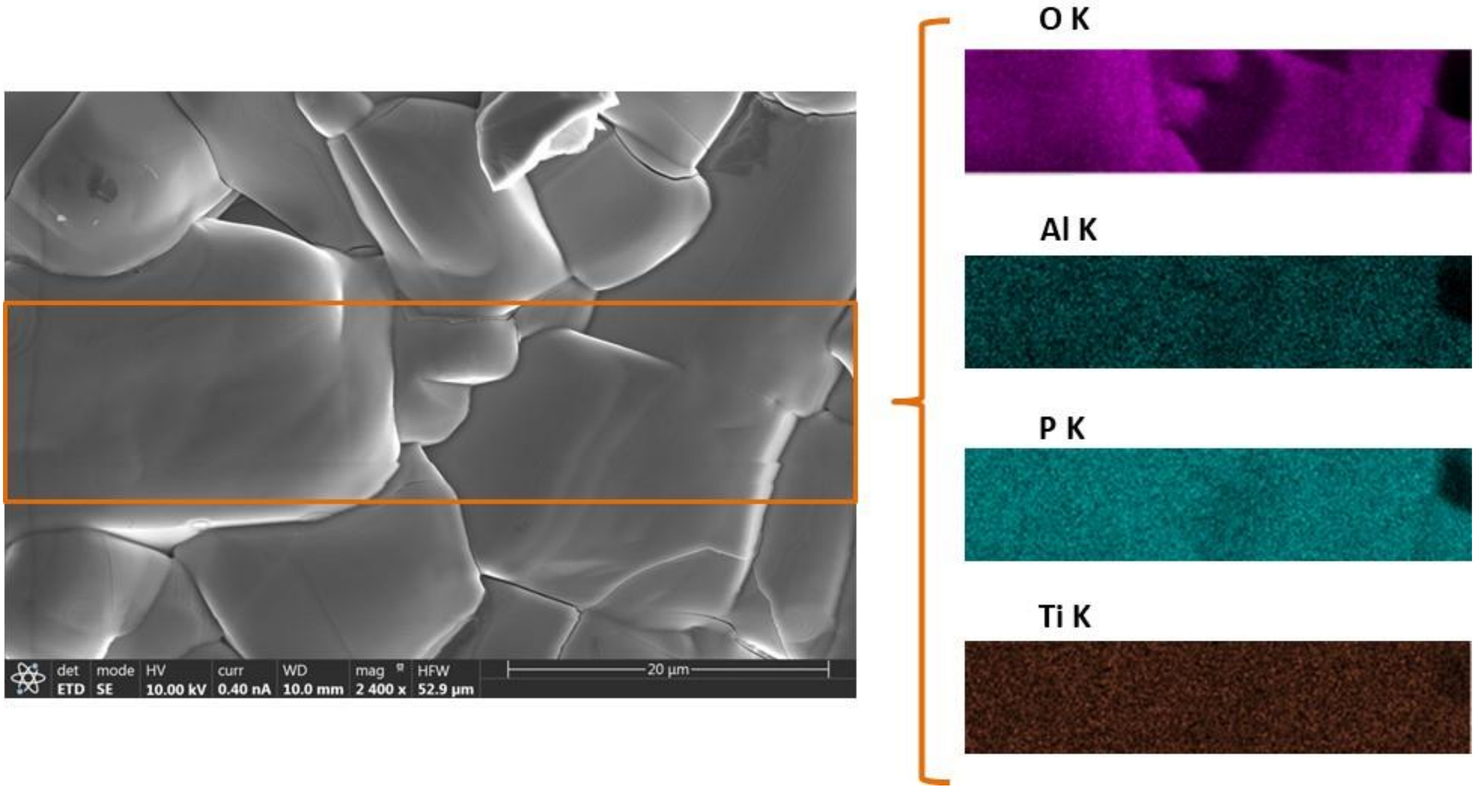

Figure S1. SEM images and mapping corresponding to the O, Al, P and Ti distribution through the cross-sectional area for the sintered LATP-0CS sample.

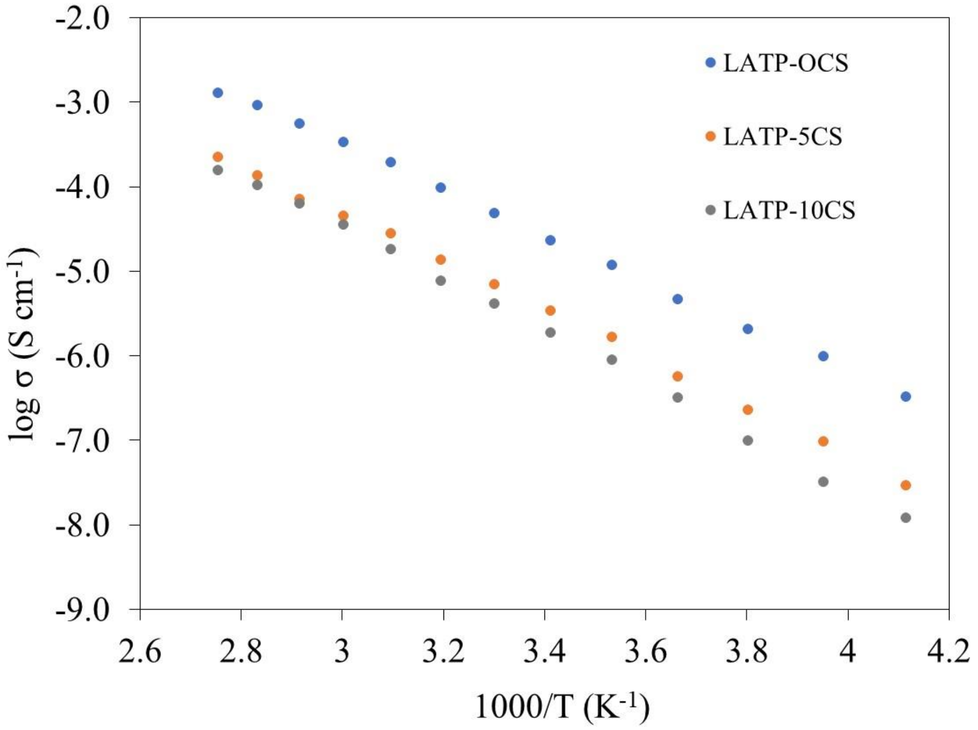

Figure S2. Arrhenius plots for LATP supports with different microstructures.

Table S1. Overall electrical conductivity at 303 and 363 K, activation energy (E_a) and the pre-exponential term of Arrhenius expression for the ceramic LATP support.

**Sample**	***A***	**E_a[eV]**	**σ [S cm^{-1}] 303K**	**σ [S cm^{-1}] 363K**
LATP-0CS	4.6	0.23	4.8x10^{-5}	1.3x10^{-3}
LATP-5CS	4.2	0.24	7.0x10^{-6}	2.2x10^{-4}
LATP-10CS	4.8	0.27	4.1x10^{-6}	1.6x10^{-4}

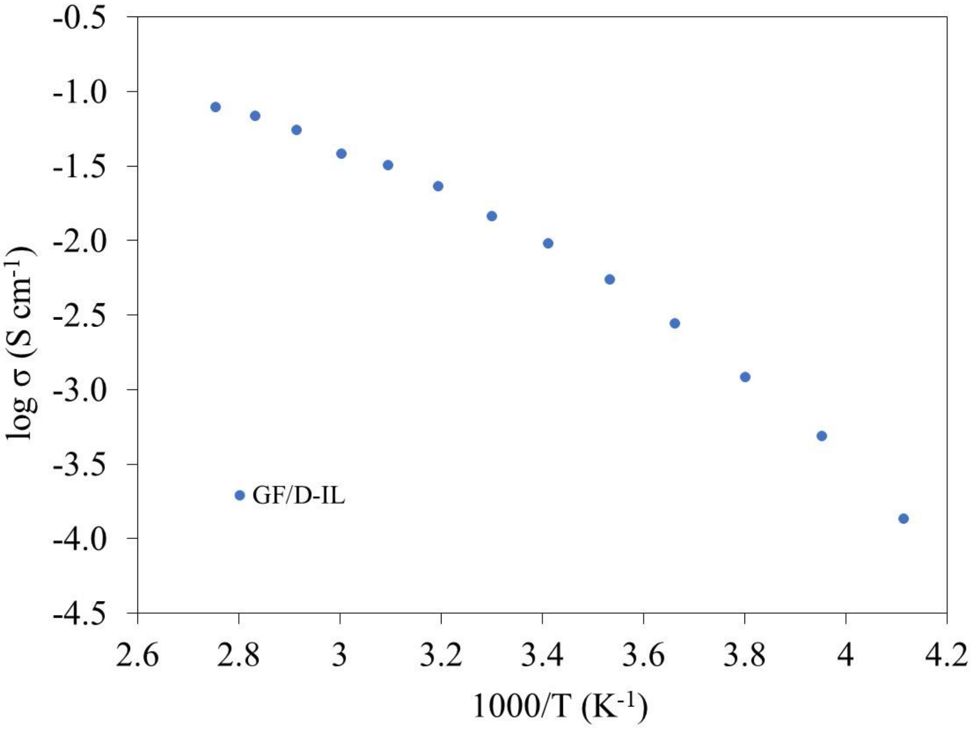

Figure S3. Arrhenius plots for Pyr_{14}TFSI-LiTFSI IL.

Table S2. Overall electrical conductivity at 303 and 363 K and parameters from VTF equations for quasi-solid state hybrid electrolytes.

**Sample**	**A [S cm^{-1} K$^{-1/2}$]**	**B [K]**	**T_0 [K]**	**E_a [eV]**	**σ [S cm^{-1}] 303K**	**σ [S cm^{-1}] 363K**
GF/D-IL	0.54	308	173	0.027	1.4x10^{-2}	7.8x10^{-2}

(a) Pyr_{14}TFSI-LTFSI-GF/D IL

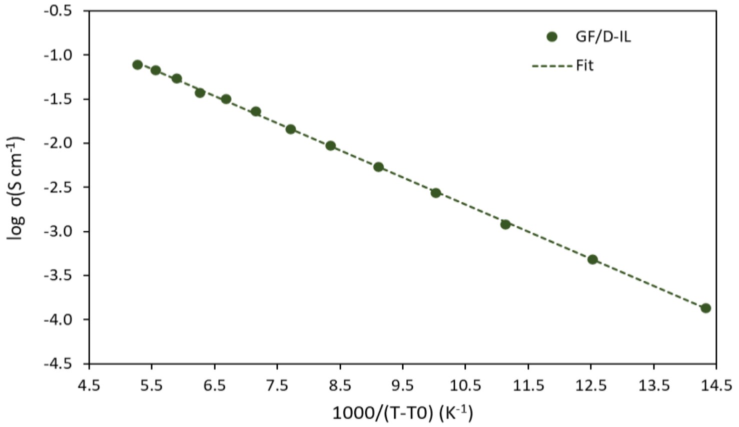

T_0 (K)	138.5
A (S cm^{-1} $K^{-1/2}$)	1.14
B (K)	595
R^2	0.9994

(b) LATP-0CS-IL

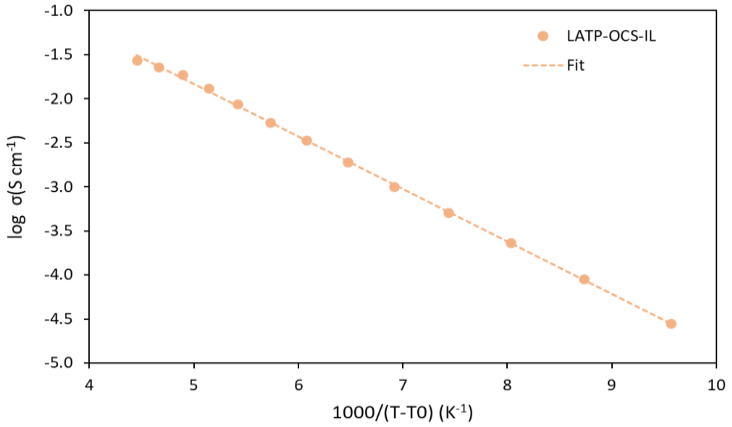

T_0 (K)	173.2
A (S cm^{-1} $K^{-1/2}$)	0.54
B (K)	308
R^2	0.9996

(c) LATP-5CS-IL

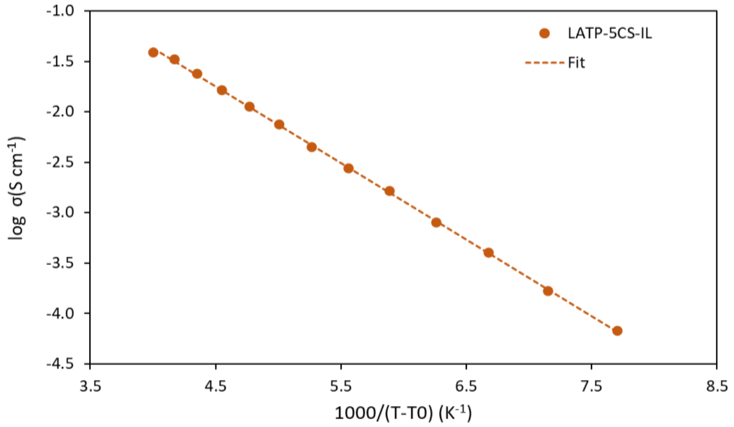

T_0 (K)	113.1
A (S cm^{-1} $K^{-1/2}$)	1.67
B (K)	759
R^2	0.9997

(d) LATP-10CS-IL

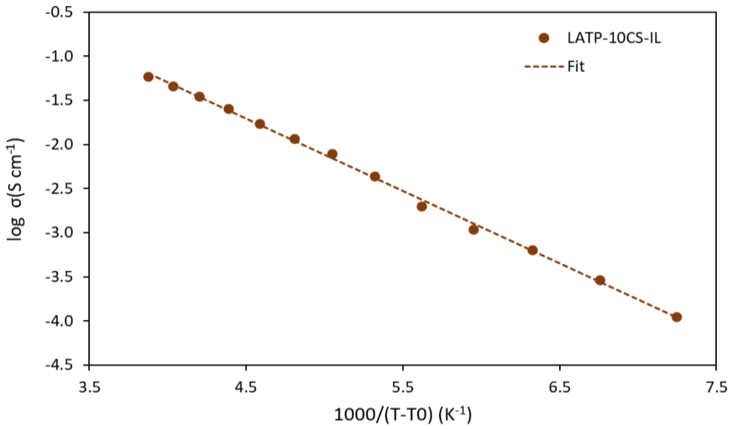

T_0 (K)	104.9
A (S cm^{-1} $K^{-1/2}$)	1.99
B (K)	821
R^2	0.9983

Figure S4. VTF fitting for (a) Pyr_{14}TFSI-LTFSI-GF/D IL, (b) LATP-0CS-IL, (c) LATP-5CS-IL and (d) LATP-10CS-IL

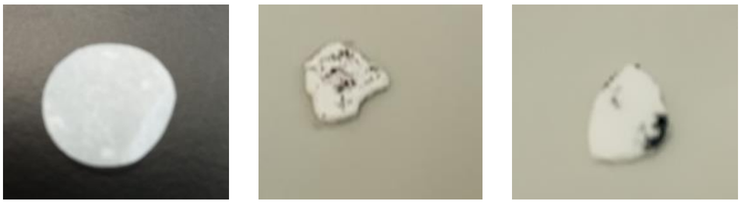

Figure S5. From left to right: pictures of glass fiber containing IL, a piece of LATP-5CS and a piece of LATP-5CS-IL after having been in contact with metallic lithium for 24 hours.
